# Thyroid-stimulating hormone levels in the normal range and incident type 2 diabetes mellitus

**DOI:** 10.1007/s00592-018-1231-y

**Published:** 2018-09-27

**Authors:** T. I. de Vries, L. J. Kappelle, Y. van der Graaf, H. W. de Valk, G. J. de Borst, H. M. Nathoe, F. L. J. Visseren, Jan Westerink, on behalf of the SMART study group

**Affiliations:** 1 Department of Vascular Medicine, University Medical Center Utrecht, P.O. Box 85500, 3508 GA Utrecht, The Netherlands; 2 Department of Neurology, University Medical Center Utrecht, Utrecht, The Netherlands; 3 Julius Center for Health Sciences and Primary Care, University Medical Center Utrecht, Utrecht, The Netherlands; 4 Department of Internal Medicine, University Medical Center Utrecht, Utrecht, The Netherlands; 5 Department of Vascular Surgery, University Medical Center Utrecht, Utrecht, The Netherlands; 6 Department of Cardiology, University Medical Center Utrecht, Utrecht, The Netherlands

**Keywords:** Thyroid-stimulating hormone, Diabetes mellitus, Prospective study, Euthyroidism

## Abstract

**Aim:**

To evaluate the relationship between thyroid-stimulating hormone (TSH) levels within the normal range and the risk of type 2 diabetes mellitus (T2DM) in a cohort of patients at high cardiovascular risk, and to perform a systematic review and meta-analysis of previous studies.

**Methods:**

We included 5542 patients without T2DM from the prospective Secondary Manifestations of ARTerial disease study with TSH levels between 0.35 and 5.0 mIU/L without anti-thyroid medication or thyroid-hormone replacement therapy. Cox regression was used to investigate the relationship between baseline plasma TSH levels and incident T2DM. MEDLINE, EMBASE, and Cochrane were searched for prospective cohorts assessing TSH and incident T2DM. Hazard ratios (HR) from included prospective cohort studies were pooled using a random-effects model.

**Results:**

In patients at high cardiovascular risk, higher plasma TSH levels in the normal range were not associated [HR 1.07 per mIU/L increase in TSH (95% confidence interval (95% CI) 0.95–1.22)] with an increased risk of T2DM, adjusted for age, sex, smoking, total and HDL cholesterol, and triglycerides. In the meta-analysis involving three prospective cohort studies, including the present study, including 29,791 participants with 1930 incident events, there was no relation between plasma TSH levels in the normal range and incident T2DM [pooled HR 1.06 (95% CI 0.99–1.14)].

**Conclusion:**

There is no apparent relation between plasma TSH levels in the normal range and incident T2DM in patients at high cardiovascular risk.

**Electronic supplementary material:**

The online version of this article (10.1007/s00592-018-1231-y) contains supplementary material, which is available to authorized users.

## Introduction

It has long been recognized that diabetes mellitus and thyroid disease, both common endocrine disorders [1, 2], are closely related [3, 4]. Type 1 diabetes mellitus and auto-immune thyroid disease are associated through common auto-immune links [5]. The underlying pathophysiological mechanisms of the repeatedly reported association between thyroid dysfunction and type 2 diabetes (T2DM) have not yet been fully elucidated [4, 6, 7]. Thyroid hormones have a large impact on glucose homeostasis [8], and both high and low thyroid hormone levels are associated with peripheral insulin resistance [9–11]. Triiodothyronine (T_3_) has been shown to play a role in the protection of pancreatic island β-cells against apoptosis [12]. Furthermore, treatment of hypothyroidism may improve insulin sensitivity [13]. Contrarily, it has been found that patients with poor glycemic control in T2DM have higher risk of subclinical hypothyroidism [14], possibly due to a stimulatory effect of higher leptin levels on the hypothalamic-pituitary-thyroid axis [15]. Thus, the association between thyroid function and T2DM is bidirectional and subject to complex and interdependent interactions.

Besides the association between thyroid dysfunction, in particular hypothyroidism, and T2DM [4, 6, 7], increasing plasma thyroid stimulating hormone (TSH) levels *within the normal range* are also associated with the prevalence of T2DM in a cross-sectional study in a general adult population in China [16]. Two longitudinal studies, from the Netherlands and Korea, show conflicting results [17–19].

As T2DM is a considerable risk factor for cardiovascular events and mortality, identifying patients at high risk for developing T2DM is important. This is especially the case for patients who are already at high risk for cardiovascular disease.

In the current study, we aim to evaluate the relationship between plasma TSH levels in the normal range and the risk of incident T2DM in a cohort of patients at high cardiovascular risk. Additionally, we performed a systematic review and meta-analysis of studies assessing the relation between plasma TSH levels in the normal range and incident T2DM in euthyroid patients.

## Methods

### Cohort study

#### Study design and participants

Data were used from patients enrolled in the Second Manifestations of ARTerial disease (SMART) study, an ongoing prospective cohort study at the University Medical Center Utrecht, the Netherlands. A detailed description of the study design has been published previously [20]. From September 1996 onwards, patients referred to our institution with clinically manifest vascular disease (coronary heart disease, cerebrovascular disease, peripheral arterial disease or abdominal aortic aneurysm) or vascular risk factors (dyslipidemia, hypertension or diabetes mellitus) were asked to participate. Written informed consent was obtained from all patients. The Medical Ethics Committee of the University Medical Center Utrecht approved the study.

**Fig. 1:**
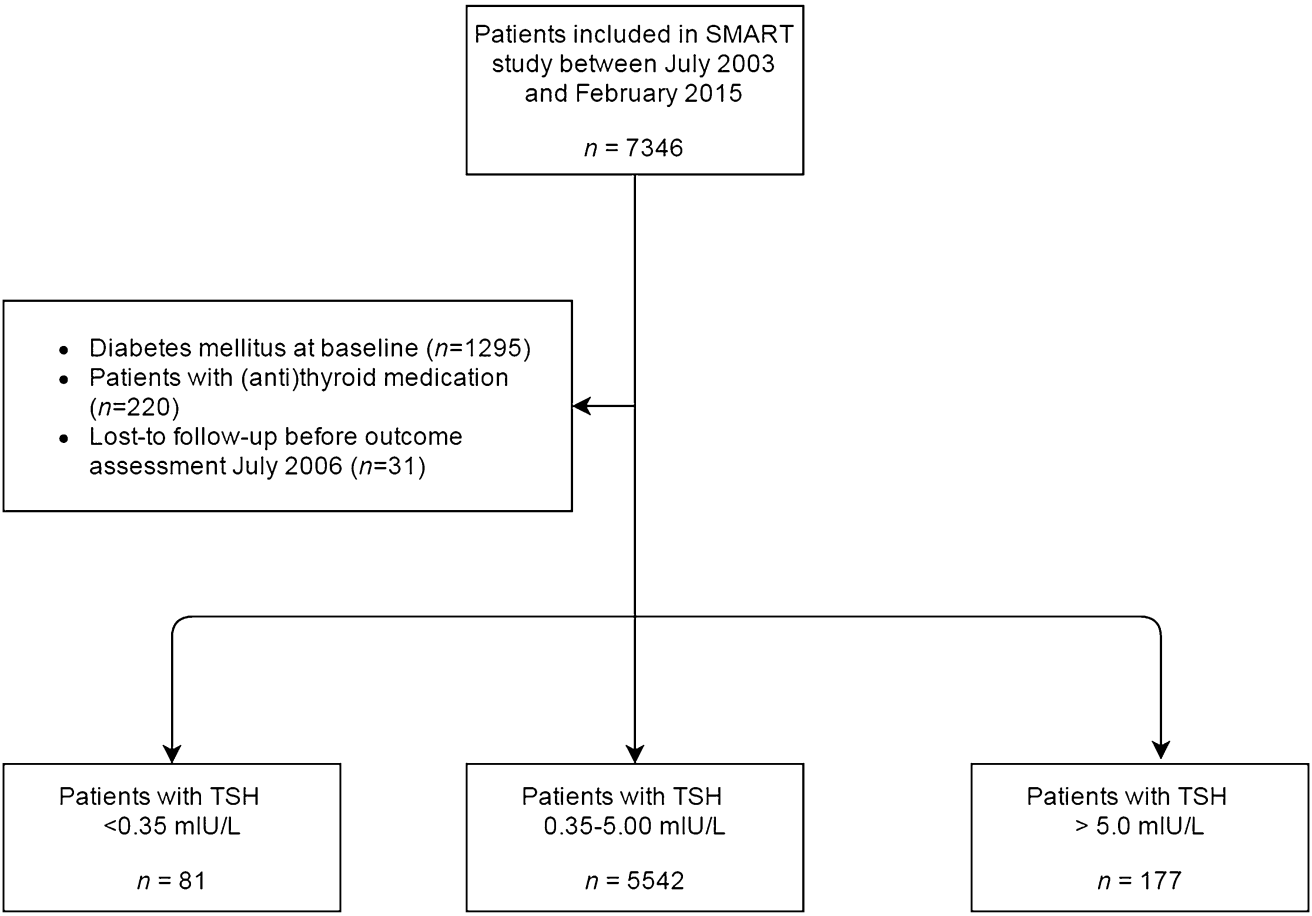
Flowchart of selection of study population

For the present study, data were used from 7346 patients included between July 2003 and February 2015, as routine measurement of TSH at baseline was added to the study protocol from July 2003 onwards. Patients with diabetes mellitus at baseline (*n* = 1295), and those receiving either thyroid hormone supplementation or anti-thyroid medication (*n* = 220) were excluded from analysis. Patients who were lost to follow-up (*n* = 31) before the assessment of incident T2DM in 2006 were also excluded. For data analyses on the relation between TSH levels and incident T2DM, patients with TSH < 0.35 mIU/L (*n* = 81) or > 5.0 mIU/L (*n* = 177) were excluded, restricting the analysis to 5542 patients with plasma TSH levels in the normal range, according to the local laboratory reference values (Fig. 1).

#### Data collection and study definitions

After inclusion, the patients underwent a standardized vascular screening protocol consisting of a health questionnaire including medical history and risk factors, physical examination and laboratory testing.

Laboratory blood testing was performed in fasting state for blood glucose, glycated hemoglobin (HbA1c), insulin levels, total cholesterol, triglycerides, high-density lipoprotein (HDL) cholesterol, and creatinine. Low-density lipoprotein (LDL) cholesterol was calculated using the Friedewald formula. The estimated glomerular filtration rate (eGFR) was calculated using the Chronic Kidney Disease Epidemiology Collaboration (CKD-EPI) formula [21]. The measurement of TSH is described in more detail in Supplementary Methods 1.

Diabetes mellitus at baseline was defined as patient-reported diagnosis of either type 1 or type 2 diabetes mellitus, use of glucose-lowering medication or insulin, or a plasma glucose concentration of ≥ 7.0 mmol/L at baseline with the commencement of glucose-lowering therapy (including diet) within 1 year after inclusion.

Metabolic syndrome was defined according to the revised National Cholesterol Education Program (NCEP-R) criteria as having at least three of the following metabolic abnormalities: waist circumference ≥ 102 cm in men or ≥ 88 cm in women, blood pressure ≥ 130 mmHg systolic and/or ≥ 85 mmHg diastolic and/or use of blood pressure-lowering agents, triglycerides ≥ 1.7 mmol/L, HDL-cholesterol < 1.0 mmol/L in men and < 1.3 mmol/L in women, fasting glucose ≥ 5.6 mmol/L [22].

#### Outcome assessment

The outcome of interest in this study was incident T2DM. As this outcome was not assessed prior to July 2006, all patients without diabetes mellitus at baseline who were included in the study before July 2006 received a questionnaire in late 2006 to assess the occurrence of T2DM after study inclusion. After 2006, the occurrence of incident T2DM was biannually assessed through questionnaires. The presence of T2DM as an outcome measure was defined as either a self-reported diagnosis and/or the use of glucose-lowering agents. Patients who reported new-onset T2DM were sent an additional questionnaire for confirmation and detailed information of the diagnosis, including the date of diagnosis, initial and current treatment, and family history of diabetes. Patients and/or their general practitioners were contacted by telephone for further information if the answers were incomplete or unclear, and non-responders were also contacted. All incident T2DM events were independently evaluated by three members of the SMART study endpoint committee. Duration of follow-up was defined as the period between study inclusion and development of incident T2DM or death from any cause, date of loss to follow-up, or the preselected date of March 1st 2015.

#### Data analyses

The baseline characteristics are described per sex-pooled quartiles of plasma TSH levels, to prevent overrepresentation of female subjects in the higher quartiles [23]. Baseline data are presented as number and percentage for categorical variables, mean ± standard deviation (SD) for normally distributed variables or median with interquartile range in case of a skewed distribution.

Cox proportional hazard models were fitted to estimate hazard ratios (HR) with 95% confidence intervals (95% CI) for plasma TSH levels as a risk factor for incident T2DM. Model I was adjusted for age and sex only, model II was additionally adjusted for smoking status, total and HDL cholesterol, and triglycerides. As measures of adiposity may be in the causal pathway [24, 25], these were not included in the primary analysis. Exploratory models were created additionally adjusting for other potential confounders; fasting serum glucose levels, BMI, SBP, the use of lipid-lowering medication, and the use of blood pressure-lowering medication. The assumption of proportionality was visually checked by plotting Schoenfeld residuals (Supplementary figure S1a). Linearity of the relation between TSH and risk of T2DM was assessed with restricted cubic splines (Supplementary figure S1b). Additionally, the same models were used to compare the plasma TSH levels as sex-pooled quartiles compared to the lowest quartile.

To investigate whether the relation between TSH and incident T2DM was modified by age, sex, or the presence of metabolic syndrome, interaction was tested between these variables and TSH for the risk of incident T2DM. A p-value of < 0.05 was considered statistically significant.

To improve statistical accuracy, missing values for potential confounders or effect modifiers [smoking status (*n* = 36), SBP (*n* = 5), total cholesterol (*n* = 15), HDL-cholesterol (*n* = 20), triglycerides (*n* = 18), and fasting serum blood glucose (*n* = 28)] were completed in the dataset by single regression imputation [26].

All statistical analysis was conducted using the statistical package R for Windows (R Foundation for Statistical Computing, Vienna, Austria).

### Systematic review and meta-analysis

The meta-analysis was conducted in accordance with the Preferred Reporting Items for Systematic Reviews and Meta-analyses (PRISMA) (Supplementary Methods 2 and Table S1). We searched PubMed, Embase and the Cochrane Library from January 1st, 1995 to October 25th, 2017, using search terms related to TSH levels and incident T2DM (Details in Supplementary Table S2). References of all eligible studies were searched for additional relevant studies. Mendeley Desktop (version 1.14) was used to merge retrieved reference and eliminate duplicates.

Studies were included that (a) identified a cohort (either as main analysis or subgroup) of participants with normal range plasma TSH levels without T2DM at baseline, (b) had a longitudinal study design, and (c) assessed the relation between baseline plasma TSH levels and the risk of incident T2DM, using measures of effect or relation (HR, odds ratio, or relative risk) with 95% CI, or enough information to allow these to be calculated. All titles and abstracts, and consequently full texts were screened according to these selection criteria. Full texts were included if they met the criteria above. The methodological quality of the included studies was assessed using the Newcastle-Ottawa scale for cohort studies (NOS) [27]. The study characteristics (name of first author, year of publication, country, study cohort, number of participants, sex distribution, mean age, duration of follow-up, number of events, reference range plasma TSH levels, and confounding variables used in the analysis) and fully adjusted HR and 95% CI were extracted from the full text of the included articles. All literature screening and data extraction was performed by two independent reviewers (TV and JW); discrepancies were resolved by discussion with a third author (FV).

The statistical analysis was performed using Review Manager (RevMan [Computer program]. Version 5.3. Copenhagen: The Nordic Cochrane Centre, The Cochrane Collaboration, 2014). The heterogeneity between the included studies was measured using the I^2^ statistic [28]. Pooled estimates were obtained with the fully adjusted HR with 95% CI of the included studies, using a random-effects model, as a random-effects model allows the overall effect to vary across studies [28, 29]. The results of the present study were also included in the pooled estimates.

## Results

### Cohort study

The baseline characteristics of the patients stratified for sex-pooled quartiles are presented in Table 1. The mean age of the study population was 56 ± 12 years, 65% of the participants were male, 27% was a current smoker at study inclusion, and 67% had a history of clinically manifest vascular disease.

**Table 1:** Patient characteristics according to sex-pooled TSH quartiles

	Quartile 1 (*n* = 1389)	Quartile 2 (*n* = 1538)	Quartile 3 (*n* = 1282)	Quartile 4 (*n* = 1333)
TSH range (mIU/L)	0.35–1.26	1.20–1.80	1.61–2.50	2.21–5.00
TSH range, men (mIU/L)	0.35–1.19	1.20–1.60	1.61–2.20	2.21–5.00
TSH range, women (mIU/L)	0.35–1.26	1.27–1.80	1.82–2.50	2.51–5.00
Male sex, *n* (%)	899 (65%)	977 (64%)	836 (65%)	877 (66%)
Age (years)	56 ± 12	55 ± 12	56 ± 12	57 ± 12
Body mass index (kg/m^2^)	27 ± 4	26 ± 4	27 ± 4	27 ± 4
Blood pressure systolic (mmHg)	140 ± 21	139 ± 22	140 ± 22	141 ± 21
Current smoker, *n* (%)	449 (32%)	475 (31%)	287 (22%)	291 (22%)
Glucose (mmol/L)	5.7 ± 0.7	5.7 ± 0.6	5.6 ± 0.7	5.7 ± 0.7
HbA1c (%)	5.6 ± 0.4	5.5 ± 0.4	5.6 ± 0.4	5.6 ± 0.4
Insulin (pmol/L)	60 (42–83)	58 (42–90)	63 (42–90)	63 (42–97)
Total cholesterol (mmol/l)	4.9 ± 1.3	4.9 ± 1.3	5.0 ± 1.4	5.0 ± 1.3
HDL-cholesterol (mmol/l)	1.3 ± 0.4	1.3 ± 0.4	1.3 ± 0.4	1.3 ± 0.4
LDL-cholesterol (mmol/l)	2.9 ± 1.1	2.9 ± 1.1	3.0 ± 1.2	3.0 ± 1.2
Triglycerides (mmol/l)	1.2 (0.9–1.8)	1.2 (0.9–1.8)	1.3 (0.9–1.8)	1.3 (0.9–1.9)
eGFR (CKD-EPI, ml/min/1.73 m^2^)	82 ± 17	81 ± 18	80 ± 17	78 ± 18
Medical history				
Clinically manifest vascular disease, *n* (%)	972 (70%)	1034 (67%)	840 (66%)	879 (66%)
Coronary heart disease, *n* (%)	622 (45%)	657 (43%)	516 (40%)	535 (40%)
Cerebrovascular disease, *n* (%)	285 (21%)	303 (20%)	242 (19%)	264 (20%)
Peripheral vascular disease, *n* (%)	136 (10%)	128 (8%)	114 (9%)	131 (10%)
Abdominal aortic aneurysm, *n* (%)	63 (5%)	61 (4%)	56 (4%)	62 (5%)
Metabolic syndrome^a^, *n* (%)	620 (45%)	598 (39%)	553 (43%)	590 (44%)
Medication use				
Lipid lowering medication, *n* (%)	874 (63%)	928 (60%)	784 (61%)	792 (59%)
Blood pressure lowering medication, *n* (%)	979 (70%)	1069 (70%)	836 (65%)	895 (67%)

*TSH* thyroid stimulating hormone, *HbA1c* glycated hemoglobin A1c, *HDL* high-density lipoprotein, *LDL* low-density lipoprotein, *eGFR* estimated glomerular filtration rate, *CKD-EPI* chronic kidney disease epidemiology collaboration

^a^According to the revised criteria of the National Cholesterol Education Program

#### Plasma TSH level as a risk factor for incident T2DM

After a median follow-up of 5.5 years (interquartile range 2.9–8.3) and a total follow-up of 31,087 person-years, there were 289 cases of T2DM (incidence rate: 9.3 per 1000 person-years, 95% CI 8.3–10.4) in patients with TSH levels in the normal range. The baseline plasma TSH level did not have a significant relationship with incident T2DM (HR 1.07; 95% CI 0.95–1.22 adjusted for age, sex, current smoking, total and HDL cholesterol, and triglycerides) (Table 2). In the exploratory models, the risk estimates did not change meaningfully (data not shown), When looking at the quartiles of baseline plasma TSH levels, there was also no significant difference between quartiles, with a fully adjusted HR of 1.07 (95% CI 0.77–1.48) for the highest compared to the lowest quartile (Table 2). Age, sex, or the presence of metabolic syndrome did not significantly modify the relation between plasma TSH levels and incident T2DM (interaction *p* values 0.66, 0.73, and 0.21, respectively).

**Table 2:** HRs (95% CI) for incident type 2 diabetes (T2DM) according to baseline plasma TSH level and according to sex-pooled quartiles of baseline TSH level

	TSH as a continuous variable^a^	Quartiles of baseline TSH level			
		Quartile 1	Quartile 2	Quartile 3	Quartile 4
		0.35–1.26	1.20–1.80	1.61–2.50	2.21–5.00
	*n* = 5542	*n* = 1389	*n* = 1538	*n* = 1282	*n* = 1333
Median TSH levels (mIU/L)		0.92	1.40	2.00	2.98
Incident T2DM, *n* (%)	289 (5.2)	73 (5.3)	70 (4.6)	72 (5.6)	74 (5.6)
HR (95% CI)					
Model I	1.08 (0.95–1.22)	1 (reference)	0.89 (0.64–1.24)	1.05 (0.76–1.46)	1.08 (0.78–1.50)
Model II	1.09 (0.96–1.23)	1 (reference)	0.90 (0.65–1.25)	1.05 (0.76–1.46)	1.07 (0.77–1.48)
Model III	1.07 (0.95–1.22)	1 (reference)	0.93 (0.67–1.29)	1.08 (0.78–1.49)	1.07 (0.77–1.48)

*HR* hazard ratios, *95% CI* 95% confidence intervals

Model I, crude model; Model II, adjusted for age and sex; Model III, adjusted for age, sex, current smoking, total and HDL cholesterol, and triglycerides

^a^The hazard ratio denotes the increase in risk for incident diabetes per 1 mIU/L rise in level of TSH within the normal range (0.35–5.0 mIU/L)

### Systematic review and meta-analysis

The search initially yielded 1361 results. After screening of title, abstract and full text, 3 articles based on 2 unique studies were eligible for inclusion (Supplementary Figure S2) [17–19]. We included the 2 studies in the meta-analysis, using the most recent article of the unique studies [17, 19]. Thus, we meta-analyzed the results from 3 studies including the present study. The study characteristics and methodological quality as assessed using the NOS can be found in Table 3 and Supplementary Table S3, respectively. The three studies included a total of 29,791 participants, with a total of 1930 events of incident T2DM.

**Table 3:** Study characteristics of studies included in meta-analysis

Study author, year	Country, cohort	Participants, *n*	Incident T2DM, *n*	Mean follow-up	Incidence rate T2DM	Mean age	No. of males (%)	Reference range TSH	Adjusted variables
Chaker et al., 2016 [17]	Netherlands, Rotterdam Study	7188	685	7.9 years	12.1 per 1000 person-years	64.6 years	3553 (42%)	0.4-4.0 mIU/L	Age, sex, smoking, cohort, fasting serum glucose levels, fasting serum insulin, SBP, DBP, blood pressure lowering medication, HDL-C, BMI
Jun et al., 2017 [18]	South Korea	17,061	956	5.0 years	11.2 per 1000 person-years	50.5 years	10,318 (60%)	0.4–4.2 mIU/L	Age, sex, smoking status, use of lipid drug, HbA1c, TG, HDL-C, LDL-C, hypertension, BMI, fasting glucose, family history of T2DM
de Vries et al., 2017 (present study)	Netherlands, SMART study	5542	289	5.6 years	9.2 per 1000 person-years	56.1 years	3589 (65%)	0.35-5.0 mIU/L	Age, sex, current smoking, total and HDL-C, and TG, (fasting glucose, BMI, SBP, lipid lowering medication, blood pressure lowering medication)

*T2DM* type 2 diabetes mellitus, *TSH* thyroid stimulating hormone, *SBP* systolic blood pressure, *DBP* diastolic blood pressure, *HDL-C* high-density lipoprotein cholesterol, *LDL-C* low-density lipoprotein cholesterol, *BMI* body mass index, *HbA1c* glycated hemoglobin A1c, *TG* triglycerides

**Fig. 2:**
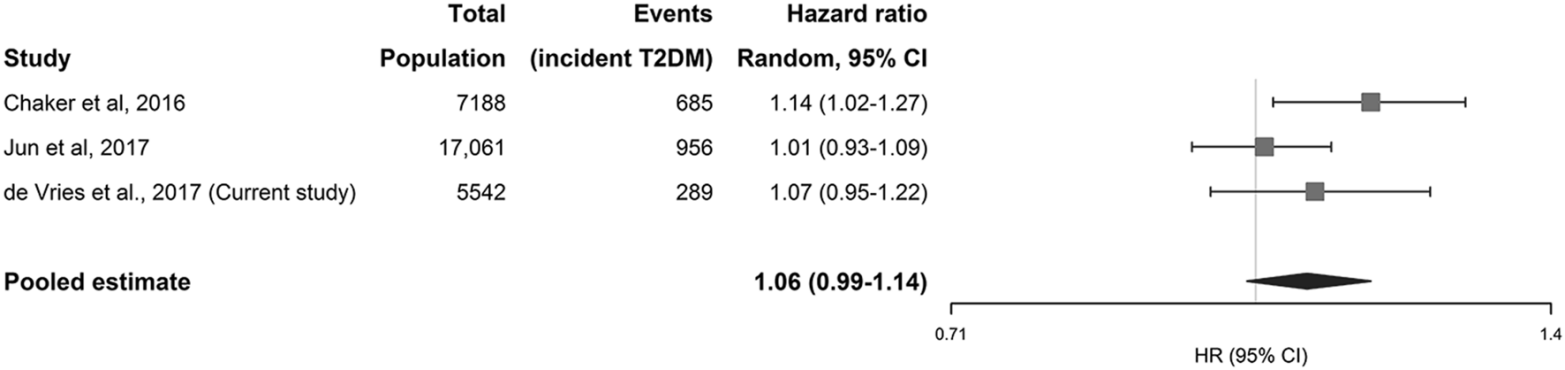
Meta-analysis of reported hazard ratios for the association between plasma TSH levels and incident T2DM, with the pooled hazard ratio

The pooled HR for the relation between continuous TSH levels within the normal range and incident T2DM was 1.06 (95% CI 0.99–1.14) (Fig. 2). Moderate statistical heterogeneity was observed, *I*^2^ = 38%.

## Discussion

In this prospective cohort study, no relation was found between plasma TSH levels within the normal range and the risk of incident T2DM in patients at high risk for cardiovascular disease. In addition, pooled results from the systematic meta-analysis of 3 studies, including the present study, showed no relation between plasma TSH levels and incident T2DM.

The results of the present study are in line with the results of the cohort study from South Korea, where the baseline plasma TSH levels in the normal range were not associated with increased risk of incident T2DM [18]. Interestingly, the authors of that study found an association between an increase in plasma TSH levels over time and incident T2DM. However, the question is whether this represents a causal relation or an opposite association, with plasma TSH levels increasing due to increasing insulin resistance in developing T2DM [30].

The results of the present study are, however, in contrast with the results from the Rotterdam Study, which found a higher risk for incident T2DM in patients with higher TSH levels in the normal range [17].

A notable difference between the studies is the used reference range for euthyroidism (0.4–4.2 mIU/L in the Korean study [19], 0.4–4.0 mIU/L in the Rotterdam Study [17], and 0.35–5.0 mIU/L in the present study). Of note is that the reference range used in the Korean study is based on Western values [23], while a recent Korean study showed that the reference value of plasma TSH in the Korean population is higher (0.62–6.68 mIU/L in 6564 participants) [31]. To exclude the possibility that the different reference range in the present study explains the differences in the results, we repeated our analyses using the reference value of 0.4–4.2 mIU/L. This analysis did not change the risk estimates meaningfully (data not shown).

Furthermore, the domains the study populations were taken from are different. The Rotterdam Study included participants among all inhabitants aged 55 years and older in one district in the city Rotterdam. The Korean Study included participants among people participating in a yearly health check-up program in a single center. Thus, these populations are very different from the population in our study, which consists of patients with high cardiovascular risk, referred to a secondary and tertiary health care center. The Rotterdam Study has a higher proportion of women in the study population (58%) compared with the Korean study (40%) and the present study (35%). In the general population women on average have higher plasma TSH values than men [23]. Furthermore, the Rotterdam Study had a higher mean age (65 years), compared to the Korean study (51 years) and the present study (56 years). It is well-recognized that plasma TSH levels increase with age [23]. However, the primary analysis was adjusted for both age and sex, and interaction analysis in both the present study and the Rotterdam Study showed that age and sex were not important effect modifiers [17]. Additionally, compared to the Korean study, the participants in the present study used more lipid lowering medication, independently a risk factor for incident T2DM (data not available for the Rotterdam Study) [32]. However, adjustments for lipid lowering medication in an exploratory analysis in the present study had no significant effect on the risk estimate. Therefore, it is not likely that these differences between the study populations fully explain the differences in study results. Finally, the present study consists mostly of participants from Caucasian descent, whereas the participants from the Korean study are mainly from Asian descent. There are differences between Caucasian and Asian people with regards to insulin resistance and diabetes [33], body weight distribution [34], and reference TSH values [31]. The ethnicity of the patients of the Rotterdam Study has not been reported [17]. It is possible that either ethnicity or other, unknown, differences in study populations explain the differences in study estimates found in these three studies.

In a recent Mendelian randomization study of 69,033 euthyroid individuals with 12,171 cases of T2DM, no evidence for a causal relation was found between 20 genetic variants for TSH levels, and 4 variants for free thyroid hormone (fT4) levels, and insulin resistance and T2DM (Odds ratio 0.91 per SD TSH increase; 95% CI 0.78–1.07) [35]. As Mendelian randomization studies are at a low risk of confounding and reverse causality, it is a good method to ascertain causality of observational associations [36]. However, the selected loci only explained 5.64% of the total variation in TSH concentration and only euthyroid participants were included in the analysis. Furthermore, plasma TSH levels are in part determined by non-genetic factors [37], which are not taken into account in a Mendelian randomization study. Therefore, it is possible this study underestimates the relation between plasma TSH levels and incident T2DM.

The combined evidence from the Mendelian randomization study and the present study and meta-analysis does not indicate a causal relationship between plasma TSH levels in the normal range and the risk of incident T2DM. It is possible that the observed association between plasma TSH levels and incident T2DM in the Rotterdam Study was due to reverse causality (i.e. insulin resistance leading to higher plasma TSH levels) [38] and/or unidentified confounders. Based on this evidence, no recommendations with regards to screening of thyroid function in patients with high risk of developing T2DM are necessary.

Important to realize is that these results only apply to plasma TSH levels in the normal range. As thyroid dysfunction is associated with altered metabolic parameters [39], patients with thyroid dysfunction cannot be compared with euthyroid patients. Previous studies have reported an association between thyroid dysfunction and T2DM [40–42]. As free thyroxine levels were not available in the present study, thyroid dysfunction was not investigated in the scope of the present study, and therefore it is not possible to make any statements about the probable relation between thyroid dysfunction and incident T2DM.

Strengths of our study include the large number of individuals from a clinically highly relevant population of patients at high risk for cardiovascular events, the long follow-up, and the extensive availability of data for possible confounders and interaction analysis.

A limitation of the study is that T2DM was only registered as an endpoint after 2006 and this information was collected retrospectively for patients included before 2006. Patients who did not respond to the questionnaires sent in 2006 were considered as loss-to follow-up, which may induce bias as this loss-to follow-up may not have been random. Additionally, the first assessment of T2DM at follow-up was based on self-reported diabetes and was not confirmed by measuring plasma glucose levels or performing an oral glucose tolerance test. However, the patients reporting T2DM were sent an additional questionnaire asking for detailed information about the diagnosis, including medication use. This method may lead to an underestimation of the incidence rate of T2DM, however, there is no indication that the results with regards to the relation between TSH levels and T2DM will be biased. Furthermore, we have no data on the levels of free thyroxine. Therefore, we could not with certainty classify all participants as euthyroid. The Rotterdam Study showed an inverse relationship between fT4 and T2DM which we also could not investigate in the current study [17]. Finally, the studies in the meta-analysis have very different study populations. Based on previous literature, and the performed sensitivity analyses and adjustments, there are no important reasons to assume that the relationship between plasma TSH levels and incident T2DM would be different in different study populations. However, it is possible that there are unknown underlying reasons that influence this relationship in different study populations.

In conclusion, the results of the current prospective cohort study in patients at high cardiovascular risk and a separate meta-analysis do not indicate a causal relation between plasma TSH levels within the normal range and incident T2DM.

## Electronic supplementary material

Below is the link to the electronic supplementary material.

Supplementary material 1 (DOCX 439 KB)
