# Supplementary material for: Thyroid-stimulating hormone levels in the normal range and incident type 2 diabetes mellitus
Source: Acta Diabetol. 2018 Sep 27;56(4):431–40. doi: 10.1007/s00592-018-1231-y (PMC6420678; doi:10.1007/s00592-018-1231-y)
Supplement: Supplementary file 1 — Supplementary material 1 (DOCX 439 KB) [file 592_2018_1231_MOESM1_ESM.docx]

**Supplementary Appendix to “TSH levels in the normal range and incident type 2 diabetes mellitus”**

Table of contents

Appendix A – Supplementary methods and figures cohort study 2

Supplementary Methods 1 – Measurement of TSH 2

Supplementary Figure S1a – Proportionality assumption 3

Supplementary Figure S1b – Spline 3

Appendix B – Supplemental methods and results of the meta-analysis 4

Supplementary Methods 2 – PRISMA protocol for meta-analysis 4

Supplementary Table S1 – PRISMA 2009 checklist for meta-analyses 8

Supplementary Table S2 – Search strategy for meta-analysis 10

Supplementary Figure S2 – Study selection for meta-analysis 11

Supplementary Table S3 – Quality assessment of studies included in meta-analysis 12

**Appendix A – Supplementary methods and figures cohort study**

*Supplementary Methods 1: Measurement of TSH*

Before November 2006, TSH was quantified using a third-generation assay on a Centaur analyzer (Bayer, Germany). This analyzer had an interassay variation of 0.22 mU/l (6%), 4.5 mU/l (5.5%), 16.5 mU/l (5.2%). The functional sensitivity of the Centaur analyzer was 0.02mU/l with an interassay imprecision of 20%. Starting December 2006, TSH was measured by a third-generation assay on a DXi analyzer (Beckman Coulter, Woerden, The Netherlands), an analyzer with an interassay variation of 4–8%, and a functional sensitivity of 0.015mU/l with an interassay imprecision of 20%. Correlation between the two analyzers was *r*=0.9991 (*n*=69), with an intercept of –0.05mU/l (95%CI 0.22 to 0.12) and a slope of 1.04 (95%CI 1.029–1.052) (range 0–95 mU/L).

*Supplementary Figure S1: (A) Schoenfeld residuals plot for the proportional hazards assumption (X-axis shows the follow-up time in days, Y-axis shows the log hazard ratio for the relation between plasma TSH and incident type 2 diabetes [T2DM]); and (B) Spline for the relationship between TSH in the normal range and incident T2DM*


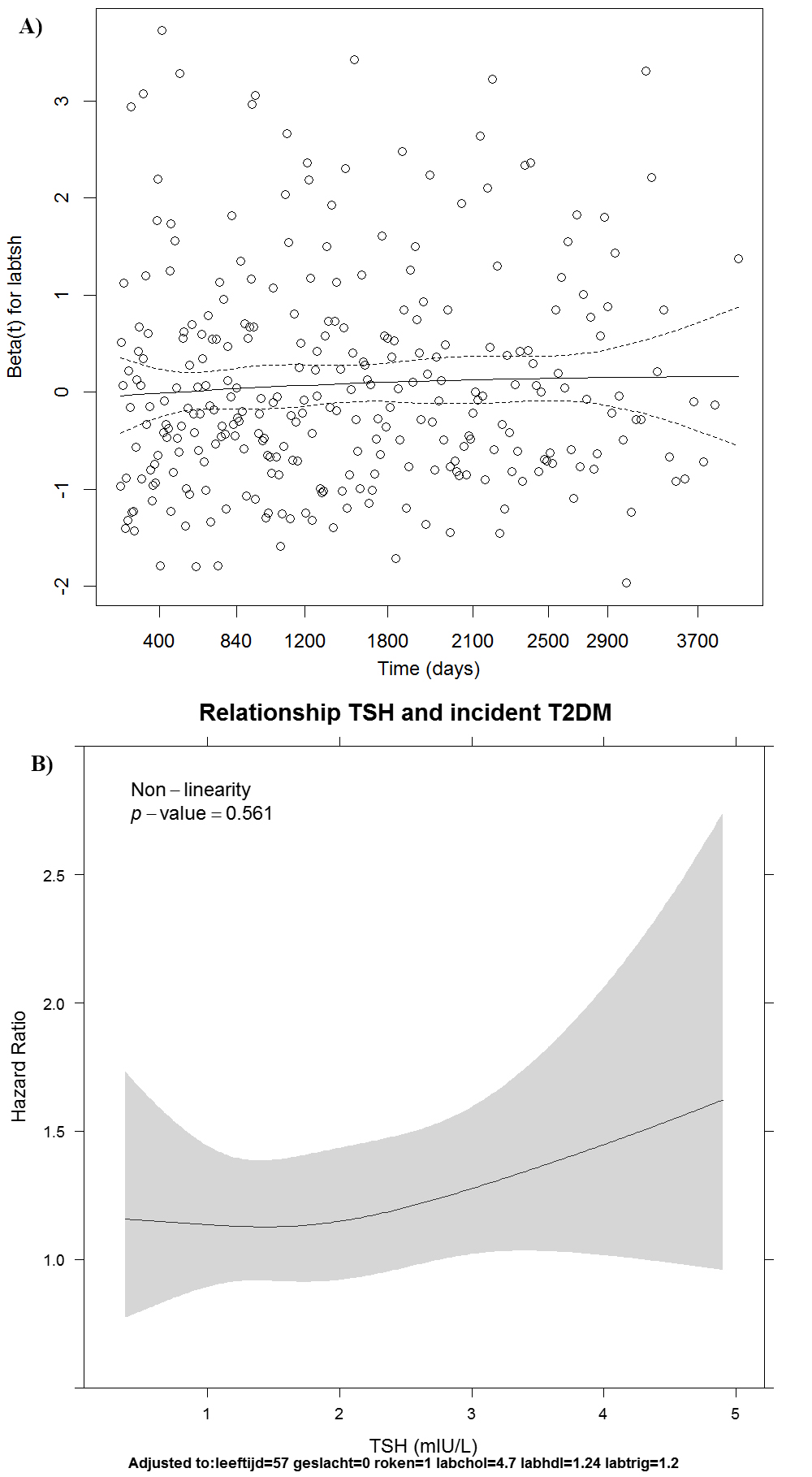


**Appendix B – Supplemental methods and results of the meta-analysis**

*Supplementary Methods 2:* PRISMA protocol for meta-analysis

**Research proposal**

**Written in line with PRISMA-P 2015 statement** June 3^rd^ 2017

**Title:** The relation between thyroid stimulating hormone levels and the risk of incident type 2 diabetes mellitus in patients with high cardiovascular risk: a cohort study and meta-analysis

**Authors:**

Tamar de Vries; Department of Vascular Medicine, University Medical Center Utrecht, Utrecht, the Netherlands; [T.I.deVries-6@umcutrecht.nl](mailto:T.I.deVries-6@umcutrecht.nl)

Prof. Frank Visseren; Department of Vascular Medicine, University Medical Center Utrecht, Utrecht, the Netherlands; [F.L.J.Visseren@umcutrecht.nl](mailto:F.L.J.Visseren@umcutrecht.nl)

Dr. Jan Westerink (corresponding author): Department of Vascular Medicine, University Medical Center Utrecht; P.O. Box 85500, 3508 GA Utrecht, the Netherlands; [J.Westerink-3@umcutrecht.nl](mailto:J.Westerink-3@umcutrecht.nl)

**Contributions:**

TV is leading protocol development, analyses and dissemination. TI and JW are first and second reviewers. FV is third reviewer. All authors will contribute to data interpretation and article drafts.

**Support:**

The SMART study was financially supported by a grant of the University Medical Center Utrecht. The funders had no role in study design, data collection and analysis, decision to publish, or preparation of the manuscript.

**INTRODUCTION**

**Rationale**

Thyroid hormones have a large impact on glucose homeostasis (1), and both high and low thyroid hormone levels are associated with peripheral insulin resistance (2–4). Triiodothyronine (T_3_) has been shown to play a role in the protection of pancreatic island β-cells against apoptosis (5), and treatment of hypothyroidism may improve insulin sensitivity (6). On the other hand, it has been found that T2DM induces higher thyroid-stimulating hormone (TSH) levels (7), possibly due to a stimulatory effect of higher leptin levels on the hypothalamic-pituitary-thyroid axis (8). Thus, the association between thyroid function and T2DM is bidirectional and subject to complex and interdependent interactions.

Besides the association between thyroid dysfunction, in particular hypothyroidism, and T2DM (9–11), increasing plasma TSH levels *within the normal range* are also associated with the prevalence of T2DM in a cross-sectional study in a general adult population in China (12). A recent longitudinal study from the Netherlands found that higher TSH levels were significantly associated with an increased risk of T2DM in 7188 euthyroid participants aged 55 years and older from the general population (13). However, studies from South Korea around the same time found no association in 17,061 euthyroid subjects from a general population (14,15).

As T2DM is a considerable risk factor for cardiovascular events and mortality, identifying patients at high risk for developing T2DM is important. This is especially the case for patients who are already at high risk for cardiovascular disease.

In the proposed study, we aim to evaluate the relationship between plasma TSH levels in the normal range and the risk of incident T2DM in a cohort of patients at high cardiovascular risk. Additionally, we will perform a meta-analysis of studies assessing the relation between plasma TSH levels in the normal range and incident T2DM.

**Objective**

To perform a meta-analysis of studies, including the proposed study, assessing the relation between plasma TSH levels in the normal range and incident type 2 diabetes.

**METHODS**

Design and methods used for this systematic review is reported in line with Preferred Reporting Items for Systematic Reviews and Meta-Analyses (PRISMA).

**Eligibility criteria**

1. Participants with normal range plasma TSH levels without T2DM at baseline
2. Longitudinal study design, observational: cohort or case-control studies
3. Determinant: baseline plasma TSH levels
4. Outcome: (risk of) incident T2DM
5. Measures of effect or relation (HR, odds ratio, or relative risk) with 95%CI, or enough information to allow these to be calculated, should be reported
6. Language: English or Dutch
7. No geographical restrictions
8. Studies published from January 1^st^ 1995 to time of systematic search

**Information sources**

PubMed, Embase and Cochrane Library. Furthermore, we will include the presently proposed cohort study.

**Search strategy in pubmed:**

(((((((((((((((TSH[Title/Abstract]) OR Thyroid-Stimulating Hormone[Title/Abstract]) OR Thyroid Stimulating Hormone[Title/Abstract]) OR thyrotropin[Title/Abstract]) OR Thyreotropin[Title/Abstract]) OR Thyroid hormone*[Title/Abstract]) OR Thyroid function*[Title/Abstract]) OR euthyroid*[Title/Abstract])) OR (("Thyrotropin"[Mesh]) OR "Thyroid Function Tests"[Mesh])))))) AND (((((((((((((((((T2DM[Title/Abstract]) OR type 2 diabetes[Title/Abstract]) OR NIDDM[Title/Abstract]) OR T2DM[Title/Abstract]) OR Maturity-Onset Diabetes[Title/Abstract]) OR Maturity Onset Diabetes[Title/Abstract]) OR Adult-Onset Diabetes[Title/Abstract]) OR Adult Onset Diabetes[Title/Abstract]) OR Non Insulin Dependent diabetes[Title/Abstract]) OR Non-Insulin Dependent diabetes[Title/Abstract]) OR Non-Insulin-Dependent diabetes[Title/Abstract]) OR Noninsulin-dependent diabetes[Title/Abstract]) OR Noninsulin Dependent diabetes[Title/Abstract]) OR slow onset diabetes[Title/Abstract]) OR slow-onset diabetes[Title/Abstract]) OR "Diabetes Mellitus, Type 2"[Mesh])))

**Study records:**

**Data management**

Records will be managed through Mendeley Desktop (version 1.14).

**Selection process**

Two reviewers (TV/JW) will search the information sources independently, and will assess identified studies for inclusion. For studies that cannot be clearly excluded on basis of its title and abstract, the full text will be reviewed independently by TV/JW. In case of disagreement, a third reviewer (FV) will mediate in the event of disagreement following discussion.

**Data collection process**

Using a standardized form, two reviewers (TV/JW) will extract the data independently. A third reviewer (FV) will independently check the data for consistency and clarity.

**Data items**

Data extracted will include the following summary data: name of first author, year of publication, country, study cohort, number of participants, sex distribution, mean age, duration of follow-up, number of outcome events, reference range plasma TSH levels, and confounding variables used in the analysis, and fully adjusted HR and 95%CI. The outcome of interest is incident type 2.

**Risk of bias in individual studies**

Risk of bias of each included trial will be independently by the same initial reviewers (TV/JW). The third reviewer will mediate in situations of disagreement. The methodological quality of the included studies will be assessed using an adaptation of the Newcastle-Ottawa scale for cohort studies (NOS), which includes: Representativeness of cohort (single center cohort or not), ascertainment of exposure (no self-report), whether it is clearly described that the outcome was not present at start, comparability of cohorts on important factors (age and gender), comparability of cohorts on additional factors (smoking status as important confounder), assessment of outcome, whether follow-up was long enough for events to occur, and the adequacy of follow-up (16).

**Data synthesis**

If studies are sufficiently homogenous in terms of design and comparator, we will conduct a meta-analysis.

The statistical analysis will be performed using Review Manager (RevMan [Computer program]. Version 5.3. Copenhagen: The Nordic Cochrane Centre, The Cochrane Collaboration, 2014), measuring the heterogeneity between the included studies using the I^2^ statistic (17). Pooled estimates will be obtained with the fully adjusted HR with 95%CI of the included studies, using a random-effects model as a random-effects model allows the overall effect to vary across studies (17,18). The results of the presently proposed cohort study will also be included in the pooled estimates.

**Meta-biases**

We will compare the fixed effect estimate against the random effects model to assess possible presence of small sample bias in the published literature. Publication bias will be assessed using a funnel plot if more than 10 studies are available.

**Confidence in cumulative evidence**

The strength of the body of evidence will be assessed based on discussion of the risk of bias in the individual studies, the risk of publication bias, differences between the included studies, and strengths and limitation of the included studies.

**References**

1. Coller FA, Huggins CB. Effect of hyperthyroidism upon diabetes mellitus: striking improvement in diabetes mellitus from thyroidectomy. Ann Surg. 1927;86(6):877–84.

2. Dimitriadis G, Baker B, Marsh H, Mandarino L, Rizza R, Bergman R, et al. Effect of thyroid hormone excess on action, secretion, and metabolism of insulin in humans. Am J Physiol. 1985;248:E593-601.

3. Lambadiari V, Mitrou P, Maratou E, Raptis AE, Tountas N, Raptis SA, et al. Thyroid hormones are positively associated with insulin resistance early in the development of type 2 diabetes. Endocrine. 2011 Feb;39(1):28–32.

4. Dimitriadis G, Mitrou P, Lambadiari V, Boutati E, Maratou E, Panagiotakos DB, et al. Insulin action in adipose tissue and muscle in hypothyroidism. J Clin Endocrinol Metab. 2006;91(12):4930–7.

5. Verga Falzacappa C, Panacchia L, Bucci B, Stigliano A, Cavallo MG, Brunetti E, et al. 3,5,3’-triiodothyronine (T3) is a survival factor for pancreatic beta-cells undergoing apoptosis. J Cell Physiol. 2006;206(2):309–21.

6. Stanicka S, Vondra K, Pelikanova T, Vlcek P, Hill M, Zamrazil V. Insulin sensitivity and counter-regulatory hormones in hypothyroidism and during thyroid hormone replacement therapy. Clin Chem Lab Med. 2005;43(7):715–20.

7. Cho JH, Kim HJ, Lee JH, Park IR, Moon JS, Yoon JS, et al. Poor glycemic control is associated with the risk of subclinical hypothyroidism in patients with type 2 diabetes mellitus. Korean J Intern Med [Internet]. 2016 Jul 1;31(4):703–11. Available from: http://kjim.org/journal/view.php?doi=10.3904/kjim.2015.198

8. Ortiga-Carvalho TM, Oliveira KJ, Soares BA, Pazos-Moura CC. The role of leptin in the regulation of TSH secretion in the fed state: in vivo and in vitro studies. J Endocrinol. 2002;174(1):121–5.

9. Han C, He X, Xia X, Li Y, Shi X, Shan Z, et al. Subclinical Hypothyroidism and Type 2 Diabetes: A Systematic Review and Meta-Analysis. PLoS One. 2015;10(8):e0135233.

10. Fleiner HF, Bjoro T, Midthjell K, Grill V, Asvold BO. Prevalence of Thyroid Dysfunction in Autoimmune and Type 2 Diabetes: The Population-Based HUNT Study in Norway. J Clin Endocrinol Metab. 2016 Feb;101(2):669–77.

11. Song F, Bao C, Deng M, Xu H, Fan M, Paillard-Borg S, et al. The prevalence and determinants of hypothyroidism in hospitalized patients with type 2 diabetes mellitus. Endocrine. 2017 Jan;55(1):179–85.

12. Gu Y, Li H, Bao X, Zhang Q, Liu L, Meng G, et al. The relationship between thyroid function and the prevalence of type 2 diabetes mellitus in euthyroid subjects. J Clin Endocrinol Metab. 2017;102(2):434–42.

13. Chaker L, Ligthart S, Korevaar TIM, Hofman A, Franco OH, Peeters RP, et al. Thyroid function and risk of type 2 diabetes: a population-based prospective cohort study. BMC Med. 2016;14(1):150.

14. Jun JE, Jee JH, Bae JC, Jin S-MS-MS-M, Hur KY, Lee M-KM-KM-KM-K, et al. Association between Changes in Thyroid Hormones and Incident Type 2 Diabetes: A Seven-Year Longitudinal Study. Thyroid. 2017;27(1):29–38.

15. Jun JE, Jin S-MS-MS-M, Jee JH, Bae JC, Hur KY, Lee M-KM-KM-KM-K, et al. TSH increment and the risk of incident type 2 diabetes mellitus in euthyroid subjects. Endocrine. 2017;55(3):944–53.

16. Wells G, Shea B, O’Connell D, Peterson J, Welch V, Losos M, et al. The Newcastle-Ottawa Scale (NOS) for assessing the quality of nonrandomised studies in meta-analyses [Internet]. Available from: http://www.ohri.ca/programs/clinical_epidemiology/oxford.asp [Accessed 11th April 2017]

17. Higgins JPT, Thompson SG, Deeks JJ, Altman DG. Measuring inconsistency in meta-analyses. Br Med J. 2003;327(7414):557–60.

18. Brockwell SE, Gordon IR. A comparison of statistical methods for meta-analysis. Stat Med. 2001;20(6):825–40.

*Supplementary Table S1.* PRISMA 2009 checklist for meta-analyses

| **Section/topic** | **#** | **Checklist item** | **Reported on page #** |
| --- | --- | --- | --- |
| **TITLE** | | |  |
| Title | 1 | Identify the report as a systematic review, meta-analysis, or both. | Page 7 |
| **ABSTRACT** | | |  |
| Structured summary | 2 | Provide a structured summary including, as applicable: background; objectives; data sources; study eligibility criteria, participants, and interventions; study appraisal and synthesis methods; results; limitations; conclusions and implications of key findings; systematic review registration number. | Page 2 |
| **INTRODUCTION** | | |  |
| Rationale | 3 | Describe the rationale for the review in the context of what is already known. | Page 3-4 |
| Objectives | 4 | Provide an explicit statement of questions being addressed with reference to participants, interventions, comparisons, outcomes, and study design (PICOS). | Page 4 |
| **METHODS** | | |  |
| Protocol and registration | 5 | Indicate if a review protocol exists, if and where it can be accessed (e.g., Web address), and, if available, provide registration information including registration number. | Available as Supplementary Methods 2 |
| Eligibility criteria | 6 | Specify study characteristics (e.g., PICOS, length of follow-up) and report characteristics (e.g., years considered, language, publication status) used as criteria for eligibility, giving rationale. | Page 7 |
| Information sources | 7 | Describe all information sources (e.g., databases with dates of coverage, contact with study authors to identify additional studies) in the search and date last searched. | Date last search October 25^th^ 2017; Page 7 |
| Search | 8 | Present full electronic search strategy for at least one database, including any limits used, such that it could be repeated. | Supplementary Table 2 |
| Study selection | 9 | State the process for selecting studies (i.e., screening, eligibility, included in systematic review, and, if applicable, included in the meta-analysis). | Page 8 |
| Data collection process | 10 | Describe method of data extraction from reports (e.g., piloted forms, independently, in duplicate) and any processes for obtaining and confirming data from investigators. | Page 8 |
| Data items | 11 | List and define all variables for which data were sought (e.g., PICOS, funding sources) and any assumptions and simplifications made. | Page 8 |
| Risk of bias in individual studies | 12 | Describe methods used for assessing risk of bias of individual studies (including specification of whether this was done at the study or outcome level), and how this information is to be used in any data synthesis. | Newcastle-Ottawa Scale; Page 8 |
| Summary measures | 13 | State the principal summary measures (e.g., risk ratio, difference in means). | HR (95%CI); Page 8 |
| Synthesis of results | 14 | Describe the methods of handling data and combining results of studies, if done, including measures of consistency (e.g., I^2^) for each meta-analysis. | RevMan 5.3; Page 8 |
| Risk of bias across studies | 15 | Specify any assessment of risk of bias that may affect the cumulative evidence (e.g., publication bias, selective reporting within studies). | - |
| Additional analyses | 16 | Describe methods of additional analyses (e.g., sensitivity or subgroup analyses, meta-regression), if done, indicating which were pre-specified. | - |
| **RESULTS** |  |  |  |
| Study selection | 17 | Give numbers of studies screened, assessed for eligibility, and included in the review, with reasons for exclusions at each stage, ideally with a flow diagram. | Supplementary Figure S2 |
| Study characteristics | 18 | For each study, present characteristics for which data were extracted (e.g., study size, PICOS, follow-up period) and provide the citations. | Table 4 |
| Risk of bias within studies | 19 | Present data on risk of bias of each study and, if available, any outcome level assessment (see item 12). | Supplementary Table S3 |
| Results of individual studies | 20 | For all outcomes considered (benefits or harms), present, for each study: (a) simple summary data for each intervention group (b) effect estimates and confidence intervals, ideally with a forest plot. | Figure 2 |
| Synthesis of results | 21 | Present results of each meta-analysis done, including confidence intervals and measures of consistency. | Figure 2; Page 7-8 |
| Risk of bias across studies | 22 | Present results of any assessment of risk of bias across studies (see Item 15). | - |
| Additional analysis | 23 | Give results of additional analyses, if done (e.g., sensitivity or subgroup analyses, meta-regression [see Item 16]). | - |
| **DISCUSSION** |  |  |  |
| Summary of evidence | 24 | Summarize the main findings including the strength of evidence for each main outcome; consider their relevance to key groups (e.g., healthcare providers, users, and policy makers). | Page 9-10 |
| Limitations | 25 | Discuss limitations at study and outcome level (e.g., risk of bias), and at review-level (e.g., incomplete retrieval of identified research, reporting bias). | Page 11 |
| Conclusions | 26 | Provide a general interpretation of the results in the context of other evidence, and implications for future research. | Page 12 |
| **FUNDING** |  |  |  |
| Funding | 27 | Describe sources of funding for the systematic review and other support (e.g., supply of data); role of funders for the systematic review. | Page 12 |

*From:* Liberati A, Altman DG, Tetzlaff J, Mulrow C, Gøtzsche PC, Ioannidis JP, Clarke M, Devereaux PJ, Kleijnen J, Moher D. The PRISMA statement for reporting systematic reviews and meta-analyses of studies that evaluate health care interventions: explanation and elaboration. PLoS Med. 2009 Jul 21;6(7):e1000100.

**Supplementary Table S2: Search strategy for meta-analysis**

| *PubMed* | (((((((((((((((TSH[Title/Abstract]) OR Thyroid-Stimulating Hormone[Title/Abstract]) OR Thyroid Stimulating Hormone[Title/Abstract]) OR thyrotropin[Title/Abstract]) OR Thyreotropin[Title/Abstract]) OR Thyroid hormone*[Title/Abstract]) OR Thyroid function*[Title/Abstract]) OR euthyroid*[Title/Abstract])) OR (("Thyrotropin"[Mesh]) OR "Thyroid Function Tests"[Mesh])))))) AND (((((((((((((((((T2DM[Title/Abstract]) OR type 2 diabetes[Title/Abstract]) OR NIDDM[Title/Abstract]) OR T2DM[Title/Abstract]) OR Maturity-Onset Diabetes[Title/Abstract]) OR Maturity Onset Diabetes[Title/Abstract]) OR Adult-Onset Diabetes[Title/Abstract]) OR Adult Onset Diabetes[Title/Abstract]) OR Non Insulin Dependent diabetes[Title/Abstract]) OR Non-Insulin Dependent diabetes[Title/Abstract]) OR Non-Insulin-Dependent diabetes[Title/Abstract]) OR Noninsulin-dependent diabetes[Title/Abstract]) OR Noninsulin Dependent diabetes[Title/Abstract]) OR slow onset diabetes[Title/Abstract]) OR slow-onset diabetes[Title/Abstract]) OR "Diabetes Mellitus, Type 2"[Mesh]))) |
| --- | --- |
| *Embase* | ('maturity-onset diabetes':ab,ti OR 'diabetes, noninsulin-dependent':ab,ti OR 'diabetes, adult-onset':ab,ti OR 'adult-onset diabetes ':ab,ti OR 'diabetes, adult onset':ab,ti OR 'diabetes, maturity-onset':ab,ti OR 'diabetes, maturity onset':ab,ti OR 'diabetes, non insulin dependent':ab,ti OR 'diabetes, non-insulin-dependent':ab,ti OR 'non-insulin-dependent diabetes ':ab,ti OR 'diabetes, noninsulin dependent':ab,ti OR 'diabetes, slow-onset':ab,ti OR 'diabetes, slow onset':ab,ti OR 'slow-onset diabetes ':ab,ti OR 'diabetes, type ii':ab,ti OR 'maturity-onset diabetes ':ab,ti OR 'maturity onset diabetes ':ab,ti OR 'type 2 diabetes ':ab,ti OR 'noninsulin-dependent diabetes ':ab,ti OR 'type 2 diabetes':ab,ti OR ‘non insulin dependent diabetes mellitus’/exp) AND  (‘TSH’:ab,ti OR ‘Thyroid-Stimulating Hormone’:ab,ti OR ‘Thyroid Stimulating Hormone’:ab,ti OR ‘thyrotropin’:ab,ti OR ‘Thyreotropin’:ab,ti OR ‘Thyroid hormone*’:ab,ti OR ‘Thyroid function*’:ab,ti OR ‘euthyroid’:ab,ti OR ‘euthyroidism’:ab,ti OR ‘euthyroidism’/exp OR 'thyrotropin'/exp) |
| *Cochrane Library* | (('maturity-onset diabetes':ab,ti or 'diabetes mellitus, noninsulin-dependent':ab,ti or 'diabetes mellitus, adult-onset':ab,ti or 'adult-onset diabetes mellitus':ab,ti or 'diabetes mellitus, adult onset':ab,ti or 'diabetes mellitus, maturity-onset':ab,ti or 'diabetes mellitus, maturity onset':ab,ti or 'diabetes mellitus, non insulin dependent':ab,ti or 'diabetes mellitus, non-insulin-dependent':ab,ti or 'non-insulin-dependent diabetes mellitus':ab,ti or 'diabetes mellitus, noninsulin dependent':ab,ti or 'diabetes mellitus, slow-onset':ab,ti or 'diabetes mellitus, slow onset':ab,ti or 'slow-onset diabetes mellitus':ab,ti or 'diabetes mellitus, stable':ab,ti or 'stable diabetes mellitus':ab,ti or 'diabetes mellitus, type ii':ab,ti or 'maturity-onset diabetes mellitus':ab,ti or 'maturity onset diabetes mellitus':ab,ti or 'type 2 diabetes mellitus':ab,ti or 'noninsulin-dependent diabetes mellitus':ab,ti or 'type 2 diabetes':ab,ti) OR (MeSH descriptor: [Diabetes Mellitus, Type 2]))  AND ((‘TSH’:ab,ti or ‘Thyroid-Stimulating Hormone’:ab,ti or ‘Thyroid Stimulating Hormone’:ab,ti or ‘thyrotropin’:ab,ti or ‘Thyreotropin’:ab,ti or ‘Thyroid hormone*’:ab,ti or ‘Thyroid function*’:ab,ti or ‘euthyroid’:ab,ti or ‘euthyroidism’:ab,ti) OR (MeSH descriptor: [Thyrotropin] OR [Thyroid Function Tests])) |

**Supplementary Figure S2.** Study selection for meta-analysis

Articles included in meta-analysis
(n = 2)

Relevant articles based on full text evaluation
(n = 3, based on 2 unique studies)

Full-text articles assessed for eligibility
(n = 7)

Records excluded
(n = 1,354)

Records screened
(n = 1,361)

Records after duplicates removed
(n = 1,361)

Additional records identified through other sources
(n = 0)

Records identified through database searching
(n = 1,783)

## Identification

## Eligibility

## Included

## Screening

Full-text articles excluded
(n = 4)

Cross-sectional study design (n = 1)

Thyroid dysfunction (n=1)

Abstract only (n=2)

**Supplementary Table S3.** Quality assessment of studies included in meta-analysis

|  | **Selection** | | | **Comparability** | | | | **Outcome** | | | **Total score (out of 8)** |
| --- | --- | --- | --- | --- | --- | --- | --- | --- | --- | --- | --- |
| **Study (first author, year)** | **Representativeness of cohort^a^** | **Ascertainment of exposure** | **Outcome was not present at start^b^** | | **Comparability of cohorts on important factor^c^** | **Comparability of cohorts on additional factor^d^** | **Assessment of outcome^e^** | | **Was follow-up long enough for events to occur?** | **Adequacy of follow-up** |  |
| Chaker et al., 2016 | 1 | 1 | 1 | | 1 | 1 | 1 | | 1 | 1 | 8 |
| Jun et al., 2017 | 0 | 1 | 0 | | 1 | 1 | 1 | | 1 | 1 | 6 |
| de Vries et al., 2017 (present study) | 0 | 1 | 1 | | 1 | 1 | 1 | | 1 | 1 | 7 |

^a^ Adapted from the NOS. As plasma TSH level is a continuous level, there is no exposed vs unexposed cohort. No point if single center cohort.

^b^ No point if relied on self-report or if method of ascertainment that outcome was not present at inclusion was not clearly described.

^c^ Must control for age and gender most importantly.

^d^ Must control for smoking status as this is an important confounder.

^e^ Assessment of type 2 diabetes must be based on the official criteria of the American Diabetes Association.
